# Prediction of the Prognosis and Treatment Responses Based on the Characteristics of Disulfidptosis-Related Genes in Patients with Cervical Squamous Cell Carcinoma and Endocervical Adenocarcinoma

**DOI:** 10.2174/0118715303374396250129111340

**Published:** 2025-02-12

**Authors:** Min Kang, Sha Jiang, Huihui Chen, Youhua Xu, Hui Mo

**Affiliations:** 1 Faculty of Chinese Medicine and State Key Laboratory of Quality Research in Chinese Medicine, Macau University of Science and Technology, Macau, 999078, China;; 2 Department of Gynecology, Zhuhai Hospital of Integrated Traditional Chinese and Western Medicine, Zhuhai, 519020, China

**Keywords:** Disulfidptosis-related gene, risk model, tumor microenvironment, cervical squamous cell carcinoma and endocervical adenocarcinoma, immune infiltration, drug sensitivity

## Abstract

**Background:**

Disulfidptosis is a new type of regulatory cell death (RCD), but the pathophysiological functions and mechanisms of disulfidptosis-related genes (DRGs) in cervical squamous cell carcinoma and endocervical adenocarcinoma (CESC) remain to be examined.

**Aims:**

This study explored the mutation status of DRGs in CESC.

**Objective:**

After analyzing the mutation profiles of DRGs in CESC, this study established a prognostic model for CESC and also explored the differences in immune infiltration (accumulation of immune system cells in tissues or organs), related enriched pathways, and drug sensitivity between high-risk and low-risk CESC groups.

**Methods:**

The Cancer Genome Atlas (TCGA) and the Gene Expression Omnibus (GEO) were accessed to source related data. The mutation profiles of DRGs in CESC were analyzed using Mutect2 software, and disulfidptosis scores were calculated by ssGSEA. WGCNA was performed to identify modular genes, which were further filtered and used to formulate a risk model by applying the survival and glmnet packages. Low- and high-risk groups of CESC patients were classified using the survminer package. GSEA was performed to conduct pathway analysis, and immune infiltration was assessed using the MCPcounter package, ESTIMATE, and TIMER algorithms. Finally, immunotherapy response and drug sensitivity were analyzed using the TIDE method and the pRRophetic package, respectively.

**Results:**

Except for *NDUFA11*, *ARL6IP5, EPM2AIP1, GBE1, RBM38, ULK4,* and *ZBTB47* were found to be the DRGs significantly mutated in CESC. The six genes were integrated to develop a RiskScore model with a relatively high Area Under the Curve (AUC) value. Significant differences between the two risk groups were determined, indicating that the model was highly reliable. Notably, the low-risk group was enriched in energy metabolism-correlated pathways, while the high-risk group was primarily enriched in immune-correlated pathways. The high-risk group showed higher immune cell activity, higher TIDE score, and more B cells than the low-risk group. Drug sensitivity study revealed that the high-risk group was more sensitive to chemotherapy drugs.

**Conclusion:**

This study provides novel insights into CESC prognosis, immunotherapy, and drug development, contributing to the clinical treatment for CESC.

## 1 INTRODUCTION

The incidence and mortality of CESC rank the eighth and ninth highest among all cancers [1]. Cervical squamous cell carcinoma is the most frequent subtype that accounts for around 75% of all CESC cases, followed by endocervical adenocarcinoma (20%) [2]. Standard treatments for CESC include surgery, concurrent chemoradiotherapy, and systemic chemotherapy [3]. CESC is primarily caused by persistent human papillomavirus (HPV) infection [4, 5]. Disease of disparity refers to a significant regional distinction in incidence and death rates between developing and developed countries [6]. Despite advances in screening, diagnostic techniques, and treatment modalities for CESC in recent years [7], the prognosis of CESC patients varies greatly. Therefore, developing prognostic models for a more accurate prediction of individual risk of occurrence, progression, and clinical outcomes in CESC is of clinical significance [8, 9].

Regulatory cell death (RCD), alternatively known as programmed cell death (PCD), is a physiological form of cellular demise [10, 11]. A recent study reported disulfidptosis as a novel type of RCD [12] that arises from NADPH deficiency under glucose-starvation conditions [13]. This might cause the overexpression of solute carrier family 7 member 11 (*SLC7A11*) in cancer cells and subsequently induce the abnormal accumulation of disulfide bonds (such as cystine) during the disruption of the actin cytoskeleton and filamentous actin (F-actin), ultimately leading to cell death [14-16]. Research indicated that compared to normal tissues, *SLC7A11* expression is elevated in various tumors and it plays a pivotal role in maintaining cellular redox balance and metabolic homeostasis [17, 18]. Furthermore, the disulfidptosis score has been validated as a predictor of prognosis in multiple cancer types [19-23]. Currently, limited studies on disulfidptosis-related genes (DRGs) necessitate a further investigation into the underlying mechanisms and potential pathophysiological functions of these genes in CESC [24].

This study explored the mechanisms of DRGs in CESC and established a DRG-based RiskScore model. To further predict the prognosis of CESC, the RiskScore was applied to assign the patients into low-risk and high-risk groups, and the differences between the two groups were explored according to their enriched pathways, immune infiltration, and drug sensitivity. The current findings could improve the understanding of the mechanisms of action of DRGs in CESC, providing a theoretical basis for prognostic assessment, immunotherapy, and drug development for CESC.

## 2 MATERIALS AND METHODS

### 2.1 Data Acquisition and Preprocessing

Somatic mutations and clinical phenotype data of CESC were downloaded from the TCGA database (https://portal.gdc.cancer.gov). Samples without complete data or lacking survival time or status were excluded and deleted while ensuring the survival time of all enrolled patients was longer than 0 day. RPKM values were calculated based on the RNA-seq data and then normalized and converted into TPM format. Subsequently, log2 transformation was performed. Finally, 291 tumor samples were used for analysis.

GSE44001 microarray dataset with survival time data was collected from the GEO database (https://www.ncbi.nlm.nih.gov/geo/). After deleting samples without clinical follow-up data or overall survival (OS), probes were transformed to symbols according to the annotation file, ultimately resulting in 300 tumor samples from GSE44001. A total of 16 DRGs, namely *RPN1*, *TLN1*, *FLNB*, *LRPPRC*, *SLC3A2*, *GYS1*, *NDUFS1*, *NCKAP1*, *OXSM*, *NUBPL*, *ACTB*, *PRDX1*, *SLC7A11*, *NDUFA11*, *MYH9*, and *FLNA,* were obtained from a previous work [13].

### 2.2 Mutation Landscape of DRGs

Mutation datasets processed by the Mutect2 software [25] from TCGA were downloaded, and the mutation characteristics of DRGs were plotted. The mutation status of the 16 DRGs in CESC was analyzed.

### 2.3 Weighted Gene Co-Expression Network Analysis (WGCNA)

The DRGs were scored by ssGSEA. Next, WGCNA was used to classify gene modules based on these signature scores [26]. Using the pickSoftThreshold function in WGCNA, the soft thresholding power β was determined. Hierarchical clustering was employed to identify gene modules under the criterion of a minimum module size of 60 genes (minModuleSize = 60). Next, the relationships between the modules and the signature scores were analyzed. Ultimately, genes in the modules with a high correlation coefficient were selected for further analysis.

### 2.4 Development and Verification of a RiskScore Model

The TCGA-CESC samples were grouped into training and testing sets at a 5:5 ratio for model construction. Utilizing the training set data, the survival package in R [27] was employed to perform univariate Cox proportional hazards regression under a threshold of *p* < 0.05 to filter prognostically significant DRGs. The candidate genes were refined by LASSO Cox regression analysis using the glmnet package in R [28] to reduce the number of genes. Key genes and the coefficients were obtained through multivariate analysis, and a Riskscore was formulated as follows:

Riskscore = Σβi × Expi

Subsequently, the survminer package [29] was utilized to determine the optimal threshold for classifying patients into low- and high-risk groups, followed by conducting a Kaplan-Meier (KM) survival analysis. Additionally, the Receiver Operating Characteristic (ROC) curve was plotted to reflect the performance of the model in the prognostic prediction.

### 2.5 Pathway Enrichment Analysis

GSEA [30] was conducted based on the candidate gene sets from the Kyoto Encyclopedia of Genes and Genomes (KEGG) database to reveal pathways involved in different biological processes (BPs) across different groups.

### 2.6 Tumor Microenvironment (TME) Analysis

The MCP-counter package [31] was employed to score 10 types of immune cells. Overall, immune microenvironment infiltration scores were calculated using ESTIMATE [32]. Furthermore, the TIMER online tool (http://cistrome .org/TIMER) was used to compute 6 immune scores based using the Timer algorithm.

### 2.7 Immunotherapy Responses and Drug Sensitivity Analysis

The TIDE method was employed to assess patients’ immunotherapy responses. Standardized transcriptome data were imported into the TIDE website (http://tide.dfci. harvard.edu/) to calculate the TIDE scores for different subgroups in the TCGA dataset, with a higher TIDE score indicating a greater likelihood of immune escape and less benefit from received immunotherapy. To assess patients’ sensitivity to widely used chemotherapy drugs, half maximal inhibitory concentration (IC50) scores for patients among different subgroups in the TCGA-CESC were computed using the pRRophetic software package [33].

### 2.8 Statistical Analysis

All the data were analyzed in R language (version 3.6.0). The Wilcoxon rank-sum test was employed to calculate the differences between two groups of continuous variables. Spearman’s method was employed to examine the association between the disulfidptosis score and stromal score, immune score, and ESTIMATE score. The survival among different groups was compared by the log-rank test. A *p-*value of < 0.05 signified a statistical significance. Sangerbox (http://sangerbox.com/) provided analytical assistance for this study [34].

## 3 RESULTS

### 3.1 Mutations in DRGs in CESC and their Association with Immunity

**Fig. (1):**
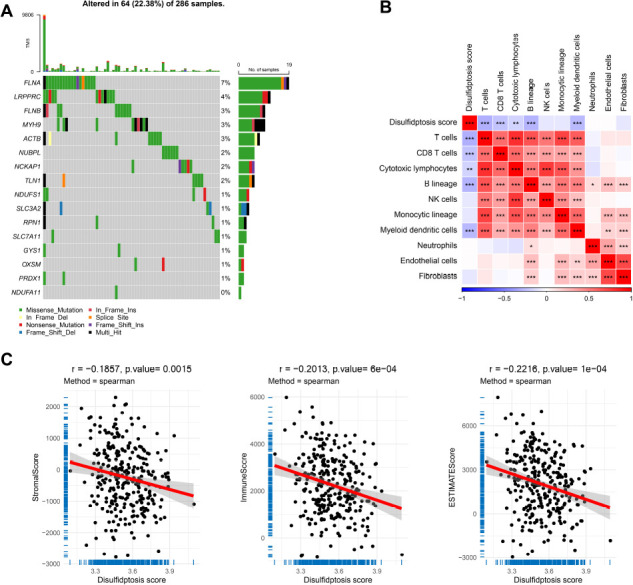
Analysis of DRG mutations and their correlation with immune cell scores. **A**: Mutation status of DRGs. **B**: Heatmap of the correlation between disulfidptosis score and the scores of 10 immune cell types assessed by MCP-counter. **C**: Scatter plot of the correlation between immune infiltration scores and disulfidptosis score evaluated by estimate. * *p* < 0.05, ** *p* < 0.01, *** *p* < 0.001, **** *p* < 0.0001.

Analysis of the mutational status of 16 DRGs in CESC revealed that apart from *NDUFA11*, all other genes exhibited varying degrees of mutations, with *FLNA* having the highest mutation frequency of 7% (Fig. **1A**). Subsequently, the relationship between the characteristics of DRGs and immunity was explored. By calculating the characteristic score of DRGs and performing correlation analysis with immune infiltration scores, it was found that the characteristic score was significantly negatively correlated with myeloid dendritic cells, T cells, B lineage, cytotoxic lymphocytes, and CD8 T cells (Fig. **1B**, *p* < 0.01). Additionally, the ESTIMATE score results demonstrated that the disulfidptosis score was significantly negatively linked to the ESTIMATE score, stromal score, and immune score (Fig. **1C**, *p* < 0.01).

### 3.2 DRGs Identified by WGCNA

**Fig. (2):**
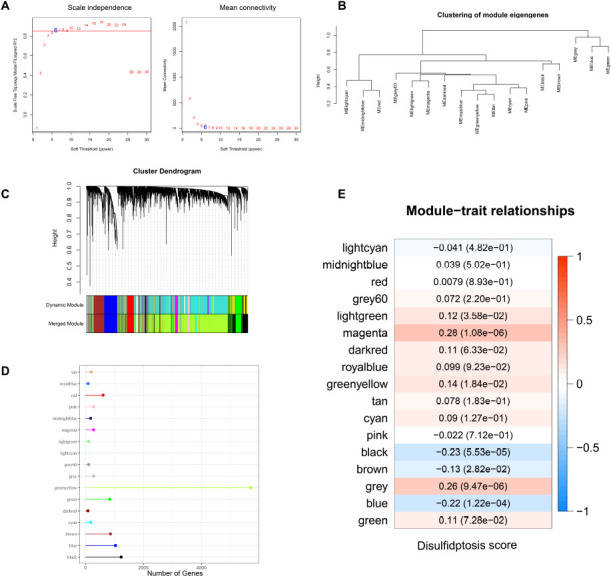
Comprehensive analysis of gene co-expression modules using WGCNA. **A**: Analysis of scale-free fitting indices for various soft-thresholding powers (β) and analysis of average connectivity for different soft-thresholding powers. **B**: Clustering dendrogram of module eigengenes. **C**: Gene dendrogram based on clustering using 1-Topological Overlap Matrix (1-TOM). **D**: Number of genes in each module. **E**: Correlation between the module eigenvectors and traits for each module.

Samples were clustered and sectioned into co-expression modules under a soft threshold β=6 to ensure a scale-free network (Figs. **2A** and **B**). Subsequently, hierarchical clustering was employed to section gene modules, resulting in 17 co-expression modules after merging (Fig. **2C**). Among them, the grey module comprised genes that could not be merged into other modules. (Fig. **2D**) shows the gene counts of each module. Further analysis detected a close correlation between the disulfidptosis score and the genes in the magenta module (Fig. **2E**).

### 3.3 Establishment of a Prognostic Model and Validation

**Fig. (3):**
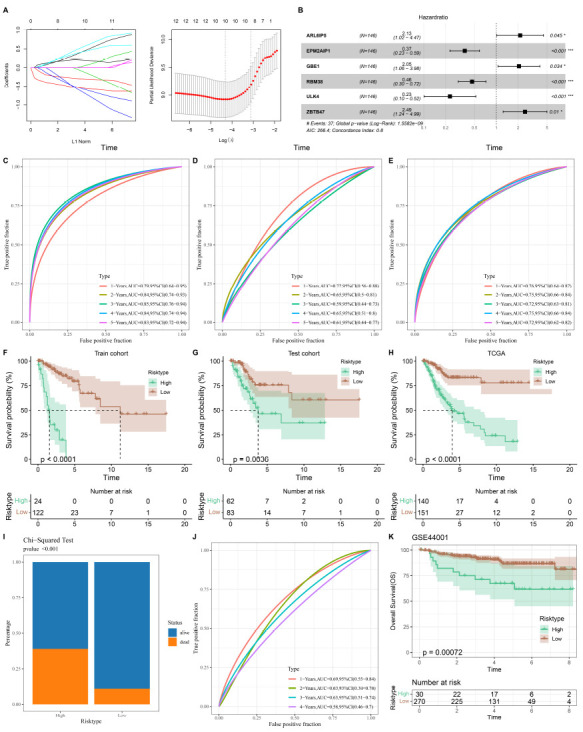
Comprehensive evaluation of a prognostic risk model. **A**: Reduction of gene number using LASSO Cox regression; **B**: Random forest plot for multifactorial analysis; **C**: ROC curve for RiskScore in the TCGA training data cohort; **D**: ROC curve for RiskScore in the TCGA validation data cohort; **E**: ROC curve for RiskScore in the TCGA cohort; **F**: KM survival curve in the TCGA training cohort; **G**: KM survival curve in the TCGA validation cohort; **H**: KM survival curve in the TCGA cohort; **I**: Survival status in the TCGA cohort; **J**: ROC curve for RiskScore in the GSE44001 cohort; **K**: KM survival curve for RiskScore in the GSE44001 cohort.

Using the data of the training set, univariate Cox proportional hazards regression was conducted on the genes in the magenta module, with a *p*-value of <0.05 applied for filtering. Subsequently, LASSO Cox regression and stepwise regression were applied to further narrow down the range of genes (Fig. **3A**), resulting in a total of 6 genes for developing a risk model (Fig. **3B**). The formula for the risk model was as follows:

Riskscore = (0.757 * ARL6IP5 - 1.002 * EPM2AIP1 + 0.719 * GBE1 - 0.769 * RBM38 - 1.472 * ULK4 + 0.912 * ZBTB47)

According to the optimal threshold of the RiskScore, the patients were classified into low-risk and high-risk groups. To test the diagnostic accuracy of the prognostic model, the AUC value of the ROC curve was calculated. The results showed high AUC values for both the training set, test set, and the TCGA cohort at various time points (Figs. **3C-E**), indicating a robust classification of the model. Notably, the OS of patients with higher RiskScore was remarkably worse than those with lower RiskScore (Figs. **3F-H**, *p* < 0.01). Compared to the low-risk group, the high-risk group had more death cases in the TCGA dataset (Fig. **3I**, *p* < 0.001). Similarly, validation of the model also showed a high AUC value in the GSE44001 dataset (Fig. **3J**). Additionally, the two risk groups manifested significant survival differences (Fig. **3K**, *p* < 0.001).

### 3.4 Differences in Enriched Pathways Between Low- and High-Risk Groups

**Fig. (4):**
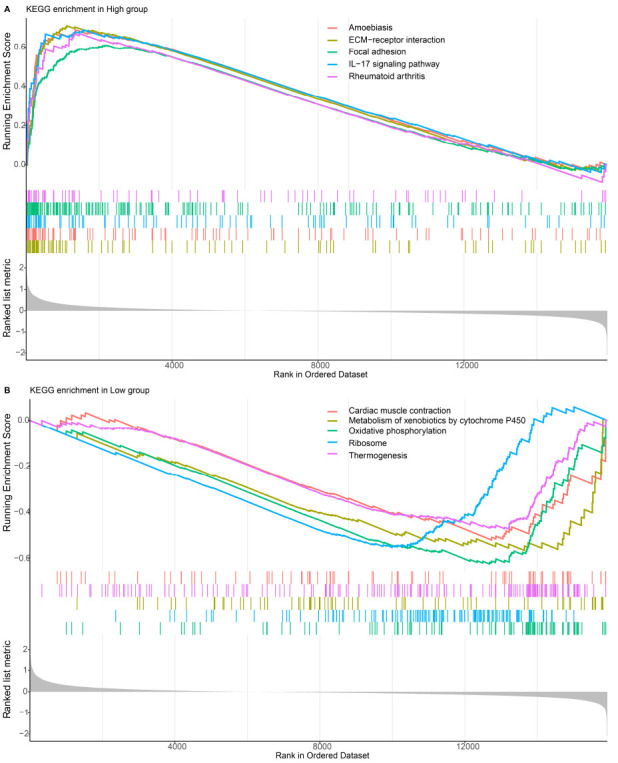
KEGG enrichment analysis for high- and low-risk groups. **A**: KEGG enrichment analysis for the high-risk group; **B**: KEGG enrichment analysis for the low-risk group.

The two risk groups manifested significant distinctions in enriched pathways. Specifically, the high-risk group displayed notable enrichment in pathways, such as amoebiasis, extracellular matrix (ECM)-receptor interaction, focal adhesion, interleukin-17 (IL-17) signaling pathway, and rheumatoid arthritis (Fig. **4A**). However, the low-risk group was enriched in pathways, including the metabolism of xenobiotics by cytochrome P450, thermogenesis, cardiac muscle contraction, ribosome, and oxidative phosphorylation (OXPHOS) (Fig. **4B**).

### 3.5 Immune Differences Between the Two Risk Groups

**Fig. (5):**
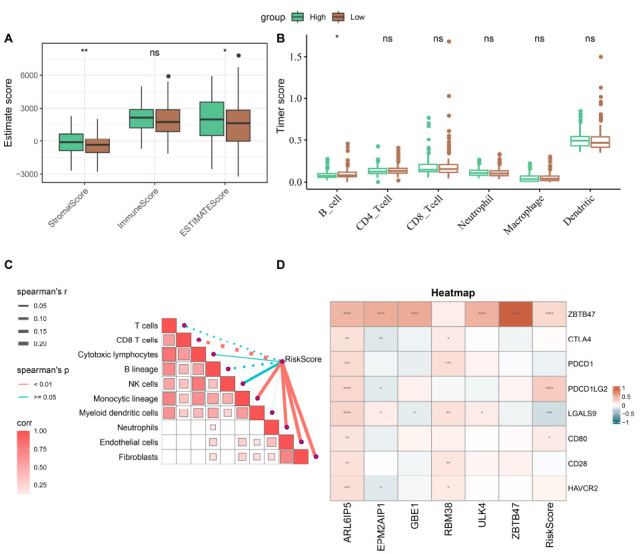
Immune infiltration and immune checkpoint analysis across high- and low-risk groups. **A**: Comparison of Stromal Score, Immune Score and ESTIMATE Score assessed by ESTIMATE across high- and low-risk groups, with * indicating *p* < 0.05, ** indicating *p* < 0.01. **B**: Immune cell (B_cell, CD4_Tcell, CD8_Tcell, Neutrophil, Macrophage and Dendritic) infiltration score calculated by the TIMER method between high- and low-risk groups, with * indicating *p* < 0.05. **C**: Correlation between MCP-counter-assessed scores of 10 immune cell types and RiskScore. **D**: Correlation between immune checkpoints, RiskScore, and prognostic genes. * *p* < 0.05, ** *p* < 0.01, *** *p* < 0.001, **** *p* < 0.0001.

Analysis of the immune microenvironment revealed that the high-risk group had noticeably higher StromalScore and ESTIMATEScore (Fig. **5A**, *p* < 0.05). The immune cell scores showed that B cells had noticeably higher scores in the low-risk group (Fig. **5B**, *p* < 0.05). Moreover, the MCPcounter analysis demonstrated that monocytic lineage, neutrophils, fibroblasts, and endothelial cells were significantly positively related to the RiskScore (Fig. **5C**, *p* < 0.01). Correlation analysis between immune checkpoint genes and the RiskScore discovered that *ZBTB47*, *PDCD1LG2*, and *CD80* were positively correlated with the RiskScore (Fig. **5D**, *p* < 0.05).

### 3.6 Drug Sensitivity Analysis

**Fig. (6):**
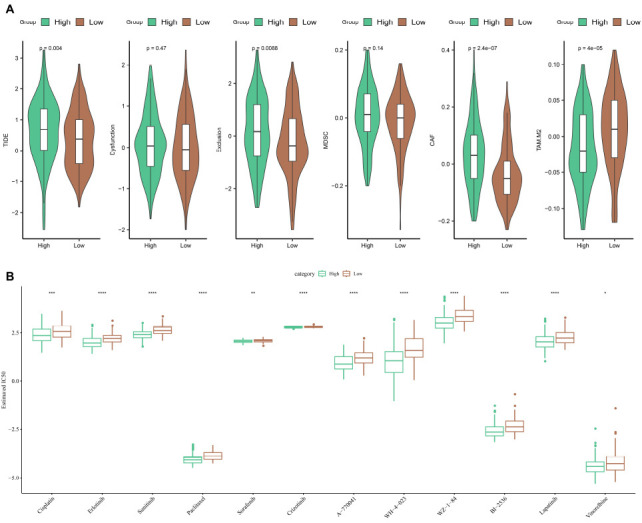
Drug sensitivity analysis between high- and low-risk groups. **A**: Differences in TIDE analysis results between high- and low-risk groups. **B**: Comparison of IC50 scores of drugs (cisplatin, erlotinib, sunitinib, paclitaxel, sorafenib, crizotinib, A−77004, WH−4−023, WZ−1−84, BI-2536, Lapatinib and Vinorelbine) between high- and low-risk groups. * *p* < 0.05, ** *p* < 0.01, *** *p* < 0.001, **** *p* < 0.0001.

Analysis of the immunotherapy responses between the two risk groups indicated that the TIDE scores in the high-risk group were notably higher in the TCGA-CESC cohort (Fig. **6A**, *p* < 0.01). The exclusion plot demonstrated that the exclusion scores were also noticeably elevated in the high-risk group, suggesting a higher level of exclusion in terms of certain immune responses or the TME (Fig. **6A**, *p* < 0.01). The high-risk group had notably higher (CAF) scores (Fig. **6A**, *p* < 0.001), indicating that CAF activity may be associated with tumor progression and alterations in the immune microenvironment within the high-risk group. However, relative to the low-risk group, M2 tumor-associated macrophages (TAM) scored significantly lower in the high-risk group (Fig. **6A**, *p* < 0.001), which indicated a lower infiltration of M2 TAMs in the high-risk group. Taken together, these results suggested that the high-risk group might have distinct immune microenvironment characteristics. Further analysis of the response to traditional chemotherapy drugs in the TCGA-CESC cohort showed that the high-risk group was sensitive to chemotherapy drugs, for instance, cisplatin, erlotinib, sunitinib, paclitaxel, A−77004, WH−4−023, and WZ−1−84 (Fig. **6B**).

## 4 DISCUSSION

CESC ranks as the fourth most common female malignancy globally [35, 36], causing over 500,000 new cases and more than 300,000 deaths each year [37]. Recent research has discovered that dithiol bond stress caused by excessive cystine accumulation in cells will lead to disulfidptosis, which can effectively inhibit the growth of malignant tumors without causing significant toxicity to normal tissues [38, 39]. The current research developed a prognostic model for CESC based on DRGs, which is as follows: RiskScore = (0.757*ARL6IP5* - 1.002*EPM2AIP1* + 0.719*GBE1* - 0.769*RBM38* - 1.472*ULK4* + 0.912*ZBTB47*). Analysis of the two risk groups revealed an increased immune cell activity and a higher sensitivity to chemotherapeutic agents in the high-risk group. The present findings contributed to the discovery of immunotherapy targets and drug development for CESC. Furthermore, this study has the potential to transform the current clinical diagnosis and treatment strategies for CESC, ultimately improving the prognosis of patients with CESC.

The current work successfully identified 6 DRGs (*ARL6IP5*, *EPM2AIP1*, *GBE1*, *RBM38*, *ULK4*, and *ZBTB47*) closely associated with CESC. Notably, these genes play pivotal roles in cell biology and tumorigenesis. *ARL6IP5* functions crucially in multiple biological processes, such as extracellular secretory protein trafficking, glutathione metabolism regulation, and tumor progression [40]. A previous study detected significantly downregulated *ARL6IP5* in CESC [41]. *EPM2AIP1* is closely related to glycogen phosphorylase [42], and its deficiency will lead to a weakened allosteric activation of glycogen synthase by glucose-6-phosphatase, thereby affecting glycogen metabolism [43]. *GBE1*, a key enzyme in the glycogen biosynthesis pathway, increases the solubility of glycogen polymers by catalyzing the formation of α-1,6-glucosidic branches from α-1,4-glucose chains, playing a critical role in maintaining the stability of glycogen structure [44]. As a member of the family of RNA-binding proteins (RBPs) containing RNA recognition motifs (RRMs) [45], *RBM38* (or *RNPC1*) can form a negative feedback regulatory loop with tumor suppressors (TS), such as members of the p53 family [46, 47], exhibiting significant inhibitory effects on various cancers [48-51]. *ULK4*, a member of the pseudokinase and Ulk kinase families, plays an indispensable role in neuronal growth and endocytosis [52, 53], and its dysfunction may be closely related to neurodegenerative diseases and tumorigenesis. *ZBTB47* belongs to the family of transcription factors containing zinc finger domains and BTB domains [54]. These proteins play decisive roles in multiple biological processes, including developmental regulation, cell differentiation, and tumorigenesis [55]. The six DRGs identified in this study may influence different stages of tumorigenesis, further validating the reliability and accuracy of the model.

According to the optimal threshold determined by the RiskScore, this study successfully categorized CESC patients into high- and low-risk groups. Further analysis showed that the high-risk group was significantly enriched in certain immune-correlated pathways, particularly the IL-17 signaling pathway and ECM-receptor interaction. Notably, ECM is primarily produced by CAFs, which is in accordance with the finding that the CAF score was higher in the high-risk group. As a crucial non-cellular component of the TME, ECM provides a supportive structure within the tumor and significantly contributes to the malignant phenotype of cancer [56, 57]. The regulation of cell proliferation, differentiation, and maintaining tissue homeostasis involves the crucial role of ECM [58, 59]. In addition, the molecular, physical, and mechanical properties of ECM regulate the migration, survival, and functions of immune cells [60]. On the other hand, IL-17, a functionally diverse and highly active pro-inflammatory cytokine, plays an indispensable role in host defense mechanisms, tissue repair processes, pathological development of inflammatory diseases, and progression of various cancers [61, 62]. The activated state of these pathways in the high-risk group may suggest more severe immune dysregulation and abnormal intercellular interactions. Meanwhile, the increased number of immune cells within the high-risk group further supported this speculation, as the proliferation of these immune cells could accelerate tumor invasion and metastasis, altering the overall landscape of the TME and significantly increasing the risk of cancer and worse prognosis.

It was also found that the B cell activity was noticeably higher in the low-risk group than in the high-risk group. B cells play regulatory roles in modulating inflammatory responses, autoimmune responses, and cancer-related immune responses [63]. B cells also effectively inhibit tumor progression through various mechanisms, such as secreting immunoglobulins, enhancing T-cell responses, and directly killing cancer cells [64]. In addition, the low-risk group was more enriched in energy metabolism pathways, including cardiac muscle contraction, OXPHOS, ribosome, metabolism of xenobiotics by cytochrome P450, and thermogenesis. Cardiac muscle contraction is related to cellular energy metabolic balance and normal physiological functions [65]. Various clinically, physiologically, and toxicologically important compounds are involved in the metabolism of xenobiotics by cytochrome P450, contributing to the maintenance of intracellular environmental stability [66]. OXPHOS produces cellular ATP, a crucial process for cellular energy production and normal cellular functions [67]. Ribosome, a large RNA-protein complex consisting of a large subunit and a small subunit, is closely related to protein synthesis [68]. Thermogenesis is significantly linked to cellular energy regulation [69]. Overall, these enriched pathways in the low-risk group helped maintain basic cellular physiological functions, metabolic homeostasis, and energy balance, which sustained a relatively stable and normal physiological state of cells in the low-risk group and also reduced the risk of cancer initiation and progression. These results indicated significant differences in cellular functional regulation and metabolic patterns between the two risk groups.

Analysis of the relationship between immune checkpoint genes and the RiskScore revealed that *ZBTB47*, *PDCD1LG2*, *CD80*, and the RiskScore were positively correlated, highlighting a close link between immune escape and the high-risk group. Immune checkpoints regulate the balance between costimulatory and coinhibitory signals, playing crucial roles in maintaining self-tolerance and modulating the magnitude and duration of T-cell responses [70]. Upregulated expression of these genes, for example, *CD80* and *PDCD1LG2,* will potentially overactivate the immunosuppressive CTLA-4 pathway and enhance the inhibitory signaling transmission between immune cells and tumorous cells, collectively suppressing the activation of immune cells, particularly T cells, and hindering their effective recognition and killing of tumor cells [71, 72]. In the high-risk group, tumor cells may utilize these high-expressed immune checkpoint genes to evade T-cell-mediated immune surveillance, preventing T cells from acquiring complete tumor antigen information and, therefore, impeding the initiation of an effective immune response. Ultimately, this will lead to a higher incidence of immune escape. A significantly higher TIDE score in the high-risk group further confirmed a greater possibility of immune escape in the high-risk patients. This result indicated that cancer cells in the high-risk group may reduce damage from immune cells through immune escape mechanisms.

However, this study also has certain limitations. Firstly, we only explored a specific patient population with CESC. Moreover, the reliance on publicly available datasets (TCGA and GEO databases) may affect the applicability of the findings. Hence, future studies are encouraged to include more CESC samples to verify whether the current model could be applied to a broader patient population. Furthermore, although this study has uncovered the potential role of DRGs in CESC, the specific mechanisms and influencing factors remained inadequately analyzed. In the future, *in vitro* and *in vivo* validation experiments should be performed for the six genes. Specifically, for *in vitro* experiments, the cell proliferation, migration and invasion, cell cycle, and apoptosis of the CESC cell line will be measured by performing CCK-8, transwell assay and wound healing, and flow cytometry in combination with transfection techniques. For *in vivo* experiments, a mouse xenograft model of the CESC tumor will be established to monitor tumor growth and collect tumor tissues for immunohistochemical analysis. The lentiviral vector could be used to regulate the gene expression of tumor cells. After validation using clinical data and clinical trials, this model could be applied to clinical practice.

## CONCLUSION

The study established a 6-gene prognosis risk model related to DRGs for CESC, providing a novel and more accurate assessment of patient outcomes. By identifying high-risk patients, clinicians can implement more aggressive or targeted therapies to improve the survival and quality of life of patients with CESC. Furthermore, our results related to the immune cell activity and chemotherapy sensitivity of high-risk patients further enrich the current understanding of CESC. These discoveries could guide the selection of immunotherapy targets and optimize treatment strategies, ultimately improving the prognosis and personalized treatment of CESC.

## Data Availability

The datasets generated and/or analyzed during the current study are available in the [GSE44001] repository (https://www.ncbi.nlm.nih.gov/geo/query/acc.cgi?acc= GSE44001).

## LIST OF ABBREVIATIONS

CESC: Cervical Squamous Cell Carcinoma and Endocervical Adenocarcinoma
HPV: Human Papillomavirus
LMIC: Low- and Middle-Income Countries
RCD: Regulatory Cell Death
PCD: Programmed Cell Death
SLC7A11: Solute Carrier Family 7 Member 11
F-actin: Filamentous Actin
DRG: Disulfidptosis-Related Gene
TCGA: The Cancer Genome Atlas
RPKM: Reads Per Kilobase of Exon Model Per Million Mapped Reads
TPM: Transcripts Per Million
GEO: Gene Expression Omnibus
OS: Overall Survival
WGCNA: Weighted Gene Co-expression Network Analysis
SsGSEA: Single-Sample Gene Set Enrichment Analysis
LASSO: Least Absolute Shrinkage and Selection Operator
KM: Kaplan-Meier
ROC: Receiver Operating Characteristic
GSEA: Gene Set Enrichment Analysis
KEGG: Kyoto Encyclopedia of Genes and Genomes
TME: Tumor Microenvironment
TIDE: Tumor Immune Dysfunction and Exclusion
IC50: Half Maximal Inhibitory Concentration
AUC: Area Under the Curve
ECM: Extracellular Matrix
IL-17: Interleukin-17
RBP: RNA-Binding Protein
RRM: RNA Recognition Motifs
TS: Tumor Suppressors
OXPHOS: Oxidative Phosphorylation
CAF: Cancer-Associated Fibroblasts
TAM: Tumor-Associated Macrophages
1-TOM: 1-Topological Overlap Matrix
